# Robotics in Pediatric Urology

**DOI:** 10.1590/S1677-5538.IBJU.2020.99.03

**Published:** 2020-02-20

**Authors:** Molly E. Fuchs, Daniel G. DaJusta

**Affiliations:** 1 Nationwide Children's Hospital ColumbusOH USA Nationwide Children's Hospital, Columbus, OH, USA

**Keywords:** Pediatrics, Robotics, Laparoscopy

## Abstract

Robotic surgery has been slow to be fully accepted in the world of pediatric urology largely because of its initial application directed towards adult use and because of the inherent high cost associated with it. However, as previously shown, it has now become the gold standard for adolescent pyeloplasty in The United States. As the adoption of robotic surgery in children has become more widespread, its use has been applied to a broader spectrum of procedures with similar success rates to standard laparoscopy. These procedures include nephrectomy, heminephrectomy, ureteral reimplantation, and ureteroureterostomy. However, it has also shown feasibility and comparable success when compared to open surgery in procedures that were previously deemed too complex to be done by standard laparoscopy. For example, bladder neck reconstruction with Mitrofanoff and Malone procedure as well as bladder augmentation. This review objective is to provide an overview of robotic surgery in pediatric urology, with a focus on the more common cases such as pyeloplasty and reimplantation as well as more complex bladder reconstruction procedures.

## INTRODUCTION

The benefits of laparoscopic surgery over open surgery are undeniable. The decrease in post-operatory pain narcotic use, blood loss and expedited recovery have helped propel the popularity of laparoscopic surgery. The learning curve has continued to be the limiting factor, particularly for more complex procedures involving intracorporeal suturing and extensive reconstruction. The popularity of laparoscopic extirpative procedures such as cholecystectomy and nephrectomy has grown to the point that they are now more common than their open counterparts. Yet, for more complex types of surgery, this trend has not seemed to hold true. Few centers were attempting laparoscopy for complex procedures such as prostatectomy and pyeloplasty and it did not seem that the laparoscopic approach would be favored over the open approach. This was largely related to the complexity of these cases and the steep learning curve associated with such procedures. It appeared that laparoscopy would not be widely adopted for complex cases until the robotic approach was introduced.

There are several primary benefits of robotic surgery over standard laparoscopic. First, 3-dimensional vision with 10 times magnification. This allows for depth perception that is lacking in standard laparoscopy. Second, the robotic Endo-Wrist instruments that allow for 7 degrees of movement freedom that far outperforms the standard laparoscopic instruments that provide limited maneuverability. Finally, in stark contrast to standard laparoscopy, the movements of the arms under the view of the camera are not inverted. As a result, the robotic platform provides more intuitive movements and proficiency is more readily acquired. The only significant limitations of robotic surgery are cost and the lack of tactile feedback, although robotic surgeons eventually overcome the lack of tactile feedback using visual cues provided by the improved optics.

As a result of these advantages, the introduction of robotics as a tool for laparoscopic surgery has allowed for many previously complex laparoscopic cases to become mainstream. Laparoscopic prostatectomy is a prime example of the robotic surgery allowing for the adoption of a minimally invasive technique. Prior to the introduction of the robotics, laparoscopic prostatectomy had been performed by a select few surgeons and because of the difficulties mastering the procedure with this technique, it failed to become adopted. In contrast, since the introduction of the robotic assisted prostatectomy, which has a more readily adoptable skill set and less steep learning curve, this technique has quickly become the gold standard in a very short period of time (1, 2). There are many similar examples of complex reconstructive surgeries that are now becoming feasible and more commonly performed due to the availability of the robotic platform.

In pediatrics, robotic pyeloplasty is the primary example of how the introduction of robotics has helped a laparoscopic technique transition from second-line therapy to the standard of care. Laparoscopic pyeloplasty was described as early as 1993 and has shown to have similar success rates as open pyeloplasty but with the added benefits of minimally invasive laparoscopic as previously discussed (3, 4). However, prior to the introduction of robotics had never been able to overtake the open surgery as the procedure of choice. A recent longitudinal evaluation of practice patterns across the US showed that in 2003, 10 years after being first described, laparoscopic pyeloplasty only accounted for <20 % of the pyeloplasties performed in patients aged 13-18 years. In contrast, ten years after robotic pyeloplasty was introduced in 2015, >80% of pyeloplasties were being performed robotically in this same age group in the United States (5). This study reinforces the sentiment that robotic assisted technique is more readily adoptable and has a more favorable learning curve compared to standard laparoscopy. Therefore, the popularity of the robotic platform continues to grow in the pediatrics. Complex reconstructive cases such as bladder neck reconstruction in neurogenic bladder, have now been performed using the robotic surgery. This technique has yet to be shown superior to its open counterpart, though this may be due to the limited number of cases performed to date.

## ROBOTIC PYELOPLASTY

As previously mentioned, the popularity of robotic pyeloplasty rose quickly and is now the gold standard for adolescent patients across the United States. In patients between 1-12 years of age, it is becoming the procedure of choice and in 2015, >40% of these patients were done robotically (5). In infants (<1 year of age), the use of robotic technique remains controversial, despite multiple reports showing the feasibility and excellent outcomes comparable to open surgery (6). The primary factors that contribute to surgeons' reluctance to adopt this technique in infants is likely the decreased intraabdominal space and the 8 mm port size of the current robotic platform. Indeed, the authors would recommend prior to attempting robotic infant pyeloplasty that the surgeon does become well familiar with performing it in bigger size patients.

One major benefit of using robotic surgery is improved ergonomics which facilitates intracorporeal suture and as a result operative times have improved when compared to standard laparoscopy. Most published series have shown a decrease in operative time with the robotic approach as well as fewer complications (7). Furthermore, the learning curve with the robot has been shown to be far shorter than with laparoscopy, thus new surgeons are able to reach the curve plateau faster (8). As a result, the number of patients exposed to the beginning of the learning curve is smaller which ultimately translates to better outcomes for patients.

Furthermore, the improved maneuverability afforded by the robot has allowed for different port positions with potential cosmetic and functional benefits. The HIdES port placement technique works extremely well for pyeloplasty irrespective of the patients' anatomy. While this type of port position does take some time to get used to, it does provide better cosmetic outcomes when compared to open surgery and standard robotic port position (9). When utilizing the HIdES technique, the surgeon must be extremely careful while placing the suprapubic camera port in a smaller patient in order to avoid a bladder injury.

The use of an internal stent has been long mention as a drawback associated with the robotic as well as laparoscopic techniques. Given that most open cases are done with an external nephroureteral stent that can be removed a week later in the office. Leaving a stent will lead to the need for an additional procedure to remove it. While this is less than ideal, several possible solutions exist. First, the stent can be placed in a retrograde fashion at the beginning of the operation and left attached to a string for later retrieval in the office. Second, given the improved optics and watertight anastomosis from running robotic suturing, a stent free pyeloplasty can be done. This later technique has been described with promising results (10). Thus, given the reported success of robotic stentless pyeloplasty, it does not seem that the stent has much impact on the overall outcome of the procedure and its use can be left to surgeons' preference.

Due to the benefits afforded by the smaller trocar incisions in laparoscopy and robotics, hospital stay continues to decrease. In most current robotic series, the hospital stay has decreased to <24 hours. With the development of Early Recovery After Surgery (ERAS) protocols, this time frame will continue to improve. To this date, only a few have reported on same day discharge after robotic pyeloplasty, but this may come to be the norm rather than the exception in the future (11).

## ROBOTIC REIMPLANTATION

Robotic ureteral reimplantation is currently a controversial topic in pediatric urology. The most common robotic technique utilized is a Lich-Gregoir extravesical approach. Multiple publications have shown that the Lich-Gregoir reimplant has similar success rates as intravesical reimplantation techniques such as the Cohen reimplant (12). Therefore, one would expect similar success rates when this technique is applied robotically. However, while the initial single-center series showed promise, a multicenter study cast serious doubt on the initial results with a success rate well below that of the open procedure (13). Furthermore, the complication rate with the robotic technique was higher when compared to open series and more than 10% of the patient required reoperation for persistent reflux or a complication. These results have caused many to reconsider the use of the robotic technique for ureteral reimplantation.

Nevertheless, two recent series, also combined results from multiple institutions to achieve a greater number of patients, showed improve success rates and a complication rate similar to open procedures (14, 15). These studies pointed out a steeper learning curve associated with this procedure as the possible cause of the initial concerning results. Another possible explanation could be related to the decreasing number of reimplantation surgeries due to the use of a more conservative approach to lower grade reflux. The above mention reasons, coupled with the fact that surgery is now only being done for a higher grade of VUR could serve as possible explanations for the initial disappointing results.

One of the main drawbacks associated with the extravesical reimplantation technique is the possibility of temporary postoperative urinary retention (14). This seems to occur at higher rates when performing a bilateral procedure as well as in patients with a prior history of voiding dysfunction. Yet, recent series have come to challenge this notion, showing other factors may play a role in the development of retention following surgery (16). Nevertheless, the surgeon needs to be aware of this complication and discuss it with the family as the patient may require intermittent catheterization usually for a short period after surgery.

While less commonly performed, laparoscopic vesicoscopic cross trigonal reimplantation has shown promise and comparable success rates (17). By insufflating and placing ports directly in the bladder, this technique avoids intraperitoneal port placement. Additionally, as this is a cross trigonal intravesical reimplantation, it carries little to no risk of urinary retention. It is a very challenging technique to master and the number of published series is limited. While it was thought that the robotic technique could make this technique more accessible, this has not been the case so far. To this date, outside of sparse case reports, large series of robotic vesicoscopic cross trigonal reimplantation has not been described in the literature (18).

While this controversy will persist, the authors believe that for an older patient with unilateral reflux robotic assisted laparoscopic extravesical reimplantation is a good surgical option. In this patient population where the bladder is usually deep in the pelvis, which can make open surgery difficult, the robotic procedure may have an edge. This may be supported by the more recent series showing similar results for the robotic technique versus open and with the prospect of faster recovery given the smaller incisions.

## ROBOTIC COMPLEX BLADDER RECONSTRUCTION

While rarely done in the past using the standard laparoscopic technique, minimally invasive complex bladder reconstruction has become a viable surgical option in pediatric patients since the introduction of the robot. The main indication for complex reconstructive surgery in pediatrics remains tied to the goal of achieving continence in patients with neurogenic bladder. This population's incontinence is usually secondary to bladder outlet incompetence, bladder overactivity, or a combination of the two problems. Bladder outlet incontinence will require a reconstructive outlet procedure most commonly coupled with a catheterizable channel while intractable bladder overactivity usually requires augmentation. Procedures such as Mitrofanoff, bladder neck reconstruction, and augmentation, are now able to be completed entirely laparoscopically with the aid of the robot and their feasibility has been clearly established. Additionally, outcomes for these surgeries performed with the robot have been comparable to their open technique counterparts. However, the overall number of patients that require these procedures is low when compared to other more common procedures such as pyeloplasty. This has hindered the ability to demonstrate the well-known benefits of robotic surgery in these procedures.

There are two strong recommendations by the authors to any surgeon undertaking robotic bladder reconstruction. First, the preoperative mechanical bowel preparation is critical. The bowel preparation's main purpose is to help increase the already limited intraabdominal space. Given that a lot of these patients have concomitant constipation related to neurogenic bowel, this can create a significant issue with intraabdominal working space if not address pre-operative. Second, these authors recommend injecting intra-detrusor Botox concomitantly with the bladder reconstruction. The bladder Botox injection has been shown to help with the post-operative bladder spasms as well as pain control (19).

Robotic-assisted technique for catheterizable channels such as appendicovesicostomy has been shown to be not just a feasible option but also has a reasonable amount of benefit. Thus far, of the bladder reconstruction procedures, robotic Mitrofanoff has the largest number of cases in the literature. A multicenter study that included 88 patients undergoing robotic Mitrofanoff with a follow up of 29.5 months, showed that the technique is reproducible across centers (20). It also demonstrated comparable complication rates and functional outcomes to previously published series of open Mitrofanoff.

Robotic bladder outlet procedure was first described by Gargollo, demonstrating the concept of feasibility (21). After the initial description, a comparison series between open and robotic cases showed similar continence outcomes and complications. The operative time was significantly longer in the robotic group. However, hospital length of stay was similar, thus there was no specific benefit to the robotic technique (22).

Robotic bladder augmentation feasibility has been well established in the literature. Additionally, functional outcomes were compared on a recent series to the open technique showing a similar increase in bladder capacity, narcotic use and complication rates between groups. The length of surgery was longer for robotic (627 vs. 265 minutes) while the length of stay was one day shorter for the robotic cohort, though this was not significant (23). Again, during the initial experience with these procedures, the usual benefits of the robotic technique have not been as evident as one would have expected.

These bladder reconstructions are complex procedures and should be performed by experienced robotic surgeons. The initial learning curve is steep, and these procedures typically take many hours longer to perform compared to their open counterparts. However, as surgeons experience grows, operative times do decrease and one cannot deny the many benefits afforded to the surgeon with the robotic technique. As the experience with these cases increases, the known benefits of laparoscopic surgery such as decreased hospital stay and narcotic use should also become evident for complex bladder reconstruction procedures.

## CONCLUSIONS

While this once seemed far in the future, robotic surgery in pediatric urology has become part of the surgeon's reality. The many benefits afforded by the robot have made laparoscopic surgery techniques accessible to surgeons. Novice laparoscopic surgeons can benefit from the shorter learning curve while skilled surgeons should be able to push the limits of what can be done laparoscopically with the application of the robotic technique. This will hopefully continue to drive towards the goal of better outcomes for the patients. There are clear benefits to using robotic surgery in pediatric urology, particularly in cases such as pyeloplasty. Its application to complex bladder reconstruction is still limited to a select few but is being applied more widely each year and this growth is limited only by the low volume of such complex procedures.
